# Detection of IgG antibody against the porcine norovirus GII.11 in human, domestic and wild animals

**DOI:** 10.3389/fmicb.2025.1567132

**Published:** 2025-06-26

**Authors:** Jiayi Xu, Huisha Du, Junxuan Yu, Ruojun Wu, Yu Zhang, Qianxin Lu, Xin Jiang, Bingwen Zeng, Tianhui Li, Qing Chen, Ying-Chun Dai

**Affiliations:** ^1^Department of Epidemiology, School of Public Health, Southern Medical University (Guangdong Provincial Key Laboratory of Tropical Disease Research), Guangzhou, Guangdong, China; ^2^Department of Pharmacology and Toxicology, Faculty of Art & Science, University of Toronto, Toronto, ON, Canada

**Keywords:** GII.11 norovirus, LISA, anthropozoonosis, cross-species transmission, porcine norovirus

## Abstract

Zoonotic diseases pose a critical threat to global public health, with noroviruses (NoVs) increasingly recognized for their potential to cross species barriers. Traditionally, NoVs were considered host-specific; however, recent evidence suggests the possibility of interspecies transmission. This study investigates the zoonotic potential of porcine NoV (PorNoV) genotype GII.11, which shares high genetic similarity with human NoVs (HuNoVs), by detecting GII.11-specific IgG antibodies in humans and various animals using a Luciferase Immunosorbent Assay (LISA). Seroprevalence was observed in humans (15.2%), pigs (49.3%), dogs (30.6%), wild rats (9.5%), and bats (65.1%), marking the first detection of GII.11 PorNoV antibodies in humans and non-swine species. Bats exhibited the highest seropositivity and antibody levels [vs. humans (*P* = 0.0011) and pigs (*P* = 0.0164)], suggesting their potential role as reservoirs. These findings provide serological evidence for anthropozoonotic transmission, challenging the paradigm of strict host specificity in NoVs. Enhanced surveillance of PorNoV in animal reservoirs and high-risk human populations is needed to mitigate zoonotic spillover risks. Further research should aim to elucidate mechanisms of transmission and the clinical significance of cross-species NoVs exposure.

## 1 Introduction

Zoonotic diseases, responsible for over 60% of emerging infectious diseases in humans, remain a formidable challenge to global public health, with profound socioeconomic and clinical impacts (Kuchipudi et al., 2021). Among these, noroviruses (NoVs), a leading cause of acute gastroenteritis worldwide, exemplify the dynamic interplay between human and animal pathogens. Classified within the family *Caliciviridae*, NoVs are non-enveloped, single-stranded RNA viruses characterized by high genetic diversity and mutation rates, traits that facilitate host adaptability and cross-species transmission (Bull et al., 2010; Wilhelm et al., 2015). Historically, NoVs were thought to exhibit strict host specificity, with human NoVs (HuNoVs) and animal NoVs (e.g., porcine NoV, PorNoV) confined to their respective hosts (Parra, 2019). However, mounting evidence now challenges this paradigm, revealing serological and molecular traces of HuNoVs in animals and vice versa (Bucardo et al., 2016; Caddy et al., 2015; Farkas et al., 2005; Nakamura et al., 2010; Widdowson et al., 2005), suggesting a bidirectional transmission risk that complicates NoVs epidemiology.

Our recent studies have identified HuNoV-specific antibodies in domestic and wild animals, including dogs, bats, and rodents (Liang et al., 2023; Wang et al., 2024), while animal NoVs, such as canine NoV (CaNoV) GVI and GIV.2, have been detected in humans (Di Martino et al., 2014; Mesquita et al., 2013). These findings underscore the potential for NoVs to transcend host barriers, yet critical gaps persist in understanding the zoonotic capacity of animal NoVs, particularly those phylogenetically proximate to HuNoVs. PorNoV, classified within genogroup II (GII), share notable genetic and antigenic similarities with HuNoVs. Despite this, seroepidemiological data on PorNoV exposure in humans and non-porcine animals remain sparse, limiting insights into their zoonotic potential.

This study focuses on GII.11 PorNoV, a strain phylogenetically clustered with HuNoVs, to investigate its cross-species transmission risk. The rationale for this selection is twofold: (1) GII.11 PorNoV exhibits the highest genetic homology to HuNoVs among animal NoVs (Saito et al., 2020), and (2) its conserved sites in P domain (Wang et al., 2007), may facilitate interspecies immune responses. While prior work has detected PorNoV in swine populations globally (Farkas et al., 2005; Mijovski et al., 2010; Silva et al., 2015), its capacity to infect humans or other animals remains unexplored. Addressing this gap is critical, as pigs-often asymptomatic carriers of PorNoV (Chao et al., 2012) -frequently interface with humans in agricultural and commercial settings, creating opportunities for spillover.

To evaluate zoonotic potential, we employed a Luciferase Immunosorbent Assay (LISA), a method validated in previous studies for its sensitivity and specificity in detecting NoVs antibodies (Liang et al., 2023; Wang et al., 2024). This approach enables high-throughput screening of GII.11 PorNoV-specific IgG in humans and diverse animal cohorts, providing serological evidence to assess cross-species exposure. By integrating epitope analysis, this study not only elucidates the seroprevalence of GII.11 PorNoV but also explores mechanisms underlying its putative host adaptability.

## 2 Materials and methods

### 2.1 Serum samples

Paired human serum samples associated with the outbreaks of GI.3 (Xie et al., 2020), GII.6 (Zhang et al., 2020), and GII.17 (Dai et al., 2017) NoV were collected from Guigang, Jinshan, and Guangzhou as described previously. Human serum samples in Jidong community-based cohort in 2018 were used for GII.11-LISA detection (Wang et al., 2021). A pilot study was conducted using a randomly selected subset of 100 serum samples to estimate the seroprevalence of GII.11 PorNoV. The preliminary analysis indicated a seroprevalence rate of 15.0%. Based on this estimate, the minimum required sample size was calculated using the formula [*n* = (*Z${}_{\alpha /2}^{2}$*) *p* (1-*p*)/*d*^2^], in which *p* = 15.0%, α = 0.05, *Z${}_{\alpha /2}^{2}$* = 1.96, and allowable error *d* = 0.05. This calculation yielded a minimum sample size of 196. To ensure robustness, a total of 250 human serum samples, representing 25% of the overall sample set (*n* = 1,000), were randomly selected for subsequent analyses. Additionally, domestic and wild serum samples were collected from pigs, dogs, bats, and rats in Guangdong and Hainan provinces, as detailed in previous studies (Liang et al., 2023; Wang et al., 2024). Specifically, negative control pig serum was procured from Shanghai Yuanye Biotechnology Co., Ltd.

### 2.2 Production of GII.11 PorNoV P protein

The cDNA fragment encoding the P proteins of the GII.11 PorNoV ch6 (Shen et al., 2012) (GenBank code: HQ392821, base 664-1644) was synthesized by BGI Shenzhen Co., Ltd., with the sequence TGCGATTGCCGTGGCGATTGCTTTTGC added to the C-terminus to enhance P protein stability. The fragment was chemically synthesized, cloned into pGEX-4T-1 expression vector (pGEX-4T-GII.11P), and transformed into *Escherichia coli* BL21(DE3) for protein expressions described in our previous study (Xie et al., 2020). The resulting GST-GII.11P fusion protein was purified using GST-Resin (Beyotime Biotechnology, China) according to the manufacturer’s instructions.

### 2.3 Generation of mouse antiserum

Four female BALB/c mice (aged 3–4 weeks) were subcutaneously immunized with GST-GII.11P fusion protein plus adjuvant at a volume ratio of 1:1, administered in four doses at 2-week intervals. An initial dose consisted of 60 μg GST-GII.11P fusion protein mixed with complete Freund’s adjuvant, followed by three booster dose of 30 μg of the protein with incomplete Freund’s adjuvant. Antiserum was isolated from the blood serum supernate and used for assessing the antigenicity identification and cross-reactivity of GII.11 NoV-LISA. Animal experimental protocol (No. D2024025) was approved by the Animal Care and Ethics Committee of Southern Medical University.

### 2.4 Construction of recombinant expression plasmid pNLF1-GII.11

The recombinant expression plasmid pNLF1-GII.11 was constructed using the Nano-Luciferase expression vector pNLF1-N (Promega, USA). The target fragments encoding P domain of GII.11 PorNoV were obtained from pGEX-4T-GII.11P using the restriction *Eco*RI and *Not*I. Subsequently, the purified fragments were inserted into the pNLF1-N vector that had been predigested with *Eco*RI and *Not*I (Takara Biotechnology Co., Ltd). Successfully verified plasmids were subsequently employed for cell transfection experiments in cultured cells.

### 2.5 Expression of GII.11 PorNoV P antigens fused with Nano-Luciferase

HEK293T cells were employed to express the Nano-Luciferase fusion protein as described previously (Liang et al., 2023; Wang et al., 2024). Briefly, HEK293T cells were seeded into 100-mm dishes in an incubator with 5% CO_2_ at 37°C. After 24 h of plating, when cell density reached 70%–90%, 5 μg of pNLF1-GII.11 plasmid was transfected into the cells using Lipofectamine 3000 (Invitrogen, USA), followed by 48 h of culture. Subsequently, the cells were washed twice with cold 0.01 M phosphate-buffered saline (PBS) and trypsinized. After being washed once with PBS, the cells were lysed at 4°C for 30 min using lysis buffer. The supernatants were obtained by centrifuging at 12,000 rpm at 4°C for 5 min and stored at −80°C.

To measure Nano-Luciferase activity in the crude cell extracts, an equal volume of Nano-Glo Luciferase assay reagent (Promega, USA) was added, and the measurement was carried out using a Tecan Infinite M200 PRO microplate luminometer.

### 2.6 Western blotting

The presence of the GII.11 PorNoV P-Nano Luciferase (GII.11P-NLuc) Fusion Protein in NLuc-antigen lysates was analyzed by Western blot (WB). After heating the samples at 100°C for 10 min, appropriate volumes of the lysate were separated on a 10% sodium dodecyl sulfate–polyacrylamide gel (SDS-PAGE). For WB analysis, proteins were electro-transferred onto a 0.22 μm PVDF membrane at 12 V for 30 min. The membrane was then blocked overnight at 4°C with 5% non-fat dry milk (NFDM) dissolved in PBS. Immune mouse antibodies against GII.11 PorNoV (dilution, 1:5,000) or rabbit anti-beta-actin monoclonal antibodies (dilution, 1:5,000) were employed as primary antibodies. Beta-actin was used as an internal control. The secondary antibodies utilized were goat HRP-conjugated anti-mouse IgG antibodies or goat HRP-conjugated anti-rabbit IgG antibodies, both diluted 1:5,000 in 3% NFDM. Peroxidase activity was detected using a Western blotting detection reagent (Amersham ECL Prime; GE Healthcare, Tokyo, Japan) and a luminometer analyzer (ChemiDoc MP Chemiluminescence Gel Imaging, Bio-Rad, USA).

### 2.7 Development of P protein-based LISA for IgG detection of GII.11 PorNoV

To detect the GII.11 PorNoV-specific IgG antibodies in serum, GII.11 NoV-LISA was implemented following the procedures (Liang et al., 2023; Wang et al., 2024). Briefly, microtiter plates were coated with protein G (5 μg/ml, 50 μl/well) in PBS and incubated overnight (14 h) at 4°C. Subsequently, after washing with PBST (PBS containing 0.05% Tween-20) and blocking with 5% NFDM in PBS for 2 h at 37°C, 50 μl of serum samples diluted 1:1,000 were added and incubated for 1 h at 37°C. After three washes, 50 μl of NLuc-antigen lysates in 2% NFDM in PBST (equivalent to 2 × 10^5^ light units) was added and incubated for 30 min at 37°C. After the final wash, 50 μl of the Nano-Glo Luciferase substrate was added to each well to measure the light units (LU) as per the manufacturer’s instructions. The cut-off value was set following recommendations from previously established LISA methodology (Liang et al., 2023; Wang et al., 2019; Wang et al., 2020; Wang et al., 2024). For GII.11 NoV-LISA, the cut-off value was also defined as twice the average LU of negative pig serum.

### 2.8 Specificity of GII.11 NoV-LISA

Specificity of GII.11 NoV-LISA was validated by investigating cross-reactivity with other enteric viruses (inter-species) and different genotypes NoVs (intra-species). For inter-species evaluation, rotavirus and aichivirus were selected. For intra-species analysis, 12 genotypes NoVs (GI.2, GI.3, GI.5, GI9, GII.2, GII4, GII.6, GII.7, GII.12, GII.13, GII.17, and GII.21) were tested. Cross-reactivity was determined using in-house hyper-immune sera generated in mice against rotavirus VP8* protein and P proteins of different genotypes NoVs, alongside rabbit sera targeting aichivirus VP1 protein, and non-immune control mice serum. All hyper-immuned and control sera were generated previously in our laboratory. All sera were measured in triplicate, and the results were presented as mean ± SD.

### 2.9 Cross-reactivity between GII.11 and GI.3, GII.6 and GII.17 sera from NoV-infected patients

To further evaluate the cross-reactivity of the GII.11 NoV-LISA in authentic infection scenarios, six paired serum samples were randomly selected for each of the GI.3, GII.6, and GII.17 NoV genotypes associated with the outbreaks (Dai et al., 2017; Xie et al., 2020; Zhang et al., 2020). Each pair consisted of one sample from the acute phase and another from the convalescent phase, with seroconversion confirmed by an antibody titer increase of at least fourfold in the convalescent phase sample compared to the acute phase sample. These selected samples were then tested to validate the assay’s performance across different NoV genotypes.

### 2.10 Detection of IgG antibody against the GII.11 PorNoV in human, domestic and wild animals

GII.11 PorNoV-specific IgG antibodies were detected using GII.11 NoV-LISA in serum samples obtained from humans, domestic animals (pigs and dogs), and wild animals (bats and rats). The evaluation included a total of 250 human, 142 pig, 72 dog, 43 bat, and 220 wild rat serum samples. Each sample was tested in duplicate to ensure reliability, with final results calculated as the mean of these measurements. Samples were classified as either positive or negative based on their reactivity. Specifically, samples exhibiting LU greater than four times the cutoff value (i.e., exceeding 4,000 LU, where the cutoff is 1,000 LU) were designated as strongly positive.

### 2.11 Epitope analysis

To further clarify the relationships between PorNoV and HuNoVs, epitope analysis was performed. All NoVs P domain sequence were collected from NCBI.^1^ Multiple sequence alignment of P capsid assessed in this study was performed using DNAMAN 9.0, with default parameters.

### 2.12 Statistical methods

Qualitative data were presented as frequency (*n*) and percentage (%). The Kruskal–Wallis test was carried out on antibody LU levels in positive samples across different species via Graph Prism 8.3.0, with Dunn’s test for multiple comparisons. Statistical significance was defined as a *P*-value < 0.05.

## 3 Results

### 3.1 Expression and identification of GII.11P-NLuc fusion protein

**FIGURE 1:**
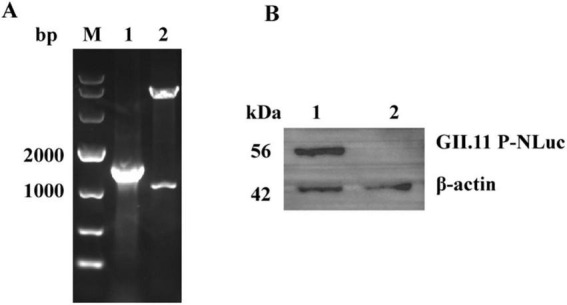
Construction and validation of the pNLF1-GII.11 recombinant plasmid. **(A)** Electrophoretic analysis of PCR amplification (lane 1) and *Eco*RI/*Not*I double-digestion (lane 2) of the recombinant pNLF1-GII.11 plasmid. Lane M: DNA marker (Takara DL10,000). **(B)** Western blot analysis of the GII.11P-NLuc fusion protein expressed in HEK293T cells. Lane 1: lysate from cells transfected with pNLF1-GII.11; lane 2: lysate from cells transfected with pNLF1-N (negative control). Proteins were probed with mouse anti-GII.11 PorNoV serum (1:5,000). A ∼55 kDa band corresponds to the expected size of the GII.11P-NLuc fusion protein. Beta-actin (42 kDa) served as a loading control.

Following the completion of PCR, double-enzyme digestion (Figure 1A) and sequencing validation, the recombinant pNLF1-GII.11 plasmid was successfully constructed, exhibiting 100% sequence identity to the anticipated sequence. Subsequently, expression of the GII.11P-NLuc fusion protein was confirmed by WB analysis (Figure 1B).

### 3.2 Specificity of GII.11 NoV-LISA

**FIGURE 2:**
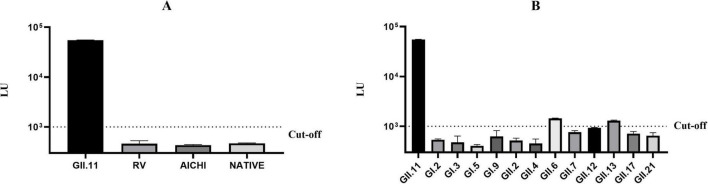
Specificity evaluation of GII.11 NoV-LISA. **(A)** Cross-reactivity testing against hyperimmune sera targeting rotavirus VP8*, aichivirus VP1 proteins. **(B)** Intra-species cross-reactivity assessment using hyperimmune sera against 12 different NoVs genotypes (GI.2, GI.3, GI.5, GI.9, GII.2, GII.4, GII.6, GII.7, GII.12, GII.13, GII.17, and GII.21). Weak cross-reactivity was observed with GII.6 (1,437 LU) and GII.13 (1,288 LU) NoVs. The dotted line indicates the assay cut-off value (1,000 LU). Data represent mean ± SD of triplicate measurements.

The cut-off value was set at 1,000 LU, twice the mean LU value of negative pig serum at 500 LU (Supplementary Table 1). The LU value of mice serum immunized with GST-GII.11P fusion protein (>50,000 LU) was significantly higher than that of the serum from mice immunized with rotavirus and aichivirus and that of the serum from non-immune mice (<500 LU), demonstrating a high specificity (Figure 2A and Supplementary Table 2). Except that GII.6 and GII.13 NoV showed weak positivity, the remainder was below the cut-off value, indicating that GII.11 NoV-LISA had the ability to distinguish GII.11 PorNoV from other NoV types (Figure 2B and Supplementary Table 2).

### 3.3 No cross-reactivity between GII.11 and GI.3, GII.6, and GII.17 sera from NoV-infected patients

Serum samples collected from patients infected with GI.3, GII.6, and GII.17 NoVs genotypes during outbreaks demonstrated seroconversion, characterized by an antibody titer in the convalescent phase that was at least fourfold (4–64 folds increase) higher than that in the acute phase for their respective genotypes (Table 1). However, none of these paired samples exhibited seroconversion in LU values when tested with the GII.11 NoV-LISA (Table 1). Specifically, the fold increase in specific antibody titers referred to antibody increase specific against GI.3, GII.6, and GII.17 NoVs. This indicated that sera from patients infected with GI.3, GII.6, and GII.17 NoVs showed no cross-reactivity with the GII.11 NoV-LISA.

**TABLE 1:** Antibody titers and folds in sera from outbreaks of GI.3, GII.6, and GII.17 norovirus genotypes and corresponding LU values detected by GII.11 NoV-LISA.

Sera of outbreak	Sample	Antibody titers of the corresponding genotype			LU value of GII.11 NoV-LISA		
		**Acute**	**Convalescent**	**Fold**	**Acute**	**Convalescent**	**Fold**
GI.3	GG01	1,000	32,000	32	491	943	2
	GG04	2,000	64,000	32	310	497	1
	GG06	4,000	16,000	4	483	538	1
	GG09	4,000	120,000	64	347	817	2
	GG11	2,000	32,000	16	469	517	1
	GG31	250	16,000	64	431	955	2
GII.6	JS03	4,000	64,000	16	819	781	1
	JS05	4,000	32,000	8	616	761	1
	JS12	500	32,000	64	709	809	1
	JS20	500	8,000	16	620	615	1
	JS22	4,000	64,000	16	564	594	1
	JS23	4,000	16,000	4	773	819	1
GII.17	GZ03	800	6,400	8	582	563	1
	GZ04	800	12,800	16	372	415	1
	GZ05	800	6,400	8	854	953	1
	GZ07	400	3,200	8	543	406	1
	GZ21	3,200	25,600	8	708	1,543	2
	GZ23	400	1,600	4	581	652	1

The fold increase refers to the ratio of antibody titers in the convalescent phase compared to those in the acute phase for paired serum samples.

### 3.4 Seroprevalence of GII.11 PorNoV-specific IgG in humans, domestic and wild animals

**FIGURE 3:**
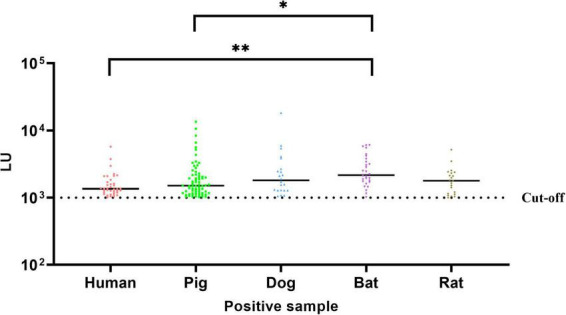
Comparative analysis of GII.11 PorNoV-specific IgG levels across species. The LU levels of GII.11 PorNoV - specific antibodies in positive sera from different species were compared using the Kruskal–Wallis test with Dunn’s multiple comparisons. Bats exhibited significantly higher LU levels compared to humans (**P = 0.0011) and pigs (*P = 0.0164). The dotted line represents the cut-off value (1,000 LU); the solid line indicates the median. Data are pooled from duplicate experiments.

GII.11 NoV-LISA was employed to detect GII.11 PorNoV specific IgG in the serum of humans, domestic (pigs and dogs) and wild (bats and rats) animals. The results showed that the positive rate of GII.11 PorNoV antibody was 15.2% in humans, 49.3% in pigs, 30.6% in dogs, and those in wild animals, such as bats and rats, were 65.1% and 9.5%, respectively (Table 2 and Supplementary Tables 3–7). Among the tested samples from humans, pigs, dogs, bats, and wild rats, the proportions of strongly positive samples were 0.04% (1/250), 4.9% (7/143), 5.6% (4/72), 14.0% (6/43), and 0.05% (1/220), respectively (Supplementary Tables 3–7). The LU levels of antibody against the GII.11 PorNoV differed significantly in positive sera among different species (Kruskal–Wallis test, *P* = 0.0020). The highest LU level was detected in bats, followed by dogs. Significant differences in LU levels were revealed between bats and both humans and pigs. Specifically, positive sera in bats exhibited higher LU levels than those in humans (*P* = 0.0011) and pigs (*P* = 0.0164) (Figure 3).

**TABLE 2:** Seroprevalence of GII.11 porcine norovirus (PorNoV)-specific IgG antibodies in humans and animals.

Genus	Location	Positive (n, %)	Negative (n, %)	Total
Human	Tangshan, Hebei	38 (15.2)	212 (84.8)	250
Pig	Guangzhou, Guangdong	70 (49.3)	72 (50.7)	142
Dog	Zhanjiang, Guangdong	22 (30.6)	50 (69.4)	72
Bat	Zhanjiang, Guangdong; Haikou, Hainan	28 (65.1)	15 (34.9)	43
Rat	Guangzhou, Guangdong	21 (9.5)	199 (90.5)	220

^1^ https://www.ncbi.nlm.nih.gov/

### 3.5 Epitope analysis of GII.11 PorNoV

A total of 35 protein sequences were selected for analysis, including 9 sequences from Genogroup GI (GI.1-GI.9), 26 Sequences from Genogroup GII (GII.1-GII.27, excluding GII.15). Epitope sites within these protein sequences were predicted using reference GII.4/2012/Sydney (GenBank: AFV08795) and GII.4/1996/USAVA387 (GenBank: AAK84679) (Chhabra et al., 2024; Yang et al., 2019).

The mean pairwise similarity of the 35 sequences was determined to be 65.3%, with 129 conserved sites (23.6%). All nine genotypes of NoV in the GI group are capable of infecting humans. Comparative analysis revealed a similarity of 71.2% between GII.11 PorNoV and the GI group, with 173 conserved sites (31.6%). The GII genotype NoVs demonstrated a closer relationship to GII.11 PorNoV, exhibiting a similarity of 74.1% and 201 conserved sites (36.7%).

**FIGURE 4:**
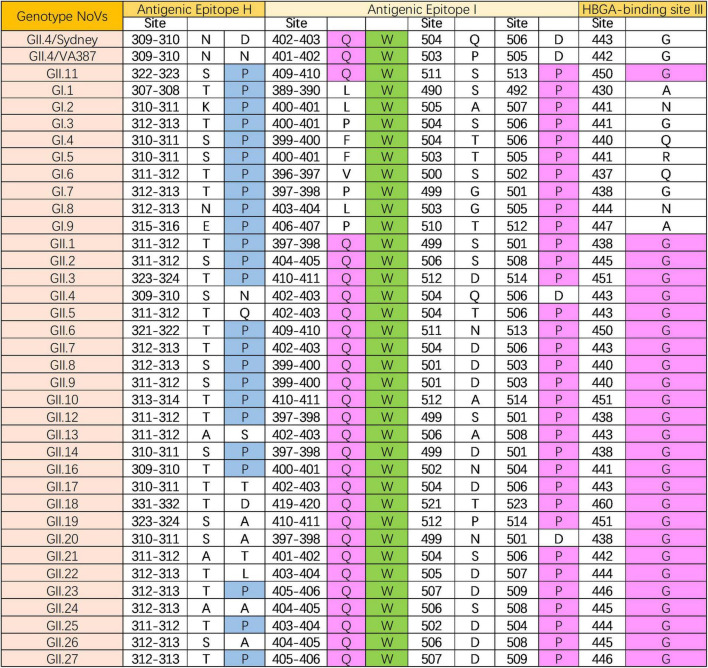
Epitope (antigenic epitope H and I, and HBGA-binding site III) analysis of GII.11 PorNoV. The letters represent the corresponding amino acid abbreviations. Green, magenta, and blue filling colors indicate homology levels of 100%, ≤75%, and ≤50%, respectively.

In addition, epitopes prediction was performed on 35 protein sequences using GII.4 HuNoV as a reference, and the conserved site W410 was identified within antigenic epitopes I. Comparison analysis of antigenic epitopes between GI NoVs and GII.11 PorNoV revealed that epitopes H(P323) and I(W410/P513) were conserved. For GII NoVs and GII.11 PorNoV, antigenic epitope I(Q409/W410) and histo-blood group antigen (HBGA) binding site (HBS) III(G450) were found to be conserved (Figure 4). Detailed information of the 35 protein sequences and the specific results of the antigenic epitope analysis can be obtained in Supplementary Figure 1.

## 4 Discussion

The emergence and resurgence of zoonotic pathogens continue to pose significant challenges to global public health, with an estimated annual burden of 2.4 billion cases of human illness and 2.7 million deaths (Rahman et al., 2020). NoV represents a notable example due to their genetic plasticity and expanding host range (Chhabra et al., 2019). Historically, NoVs were believed to exhibit strict host specificity, but accumulating evidence now challenges this paradigm, suggesting potential cross-species transmission between humans and animals (Bucardo et al., 2016; Caddy et al., 2015; Di Martino et al., 2014; Farkas et al., 2005; Liang et al., 2023; Mesquita et al., 2013; Nakamura et al., 2010; Wang et al., 2024; Widdowson et al., 2005). This study provides novel serological evidence for the zoonotic potential of GII.11 PorNoV, a strain phylogenetically proximate to HuNoVs, by detecting specific IgG antibodies in humans and non-porcine animals. To our knowledge, the present study is the first report of GII.11 PorNoV antibodies in humans and non-swine species. These findings advance our understanding of NoVs ecology and underscore the necessity of re-evaluating their host boundaries.

Our results revealed a 15.2% seroprevalence of GII.11 PorNoV-specific IgG in humans with only one strongly positive sample. This parallels prior observations of animal NoVs exposure in humans, such as the detection of CaNoV GVI antibodies in veterinarians (22.3%) and controls (5.8%) (Mesquita et al., 2013). While the detection of antibodies does not confirm active infection, it strongly suggests prior antigenic exposure, potentially through direct or indirect contact with infected animals or environmental reservoirs. The higher seropositivity in pigs (49.3%) aligns with global reports of PorNoV prevalence in swine populations, ranging from 7% to 51.8% (Farkas et al., 2005; Silva et al., 2015), reinforcing pigs as primary hosts.

However, the unexpectedly high seroprevalence in bats (65.1%) with a strongly positive rate of 14.0%, exceeding even that in pigs, warrants further attention. Bats are recognized reservoirs for numerous zoonotic viruses (Kocher et al., 2018), and their elevated antibody levels may indicate either frequent exposure or prolonged viral persistence. This raises questions about their role in maintaining and disseminating PorNoV, particularly given their mobility and proximity to human habitats. The detection of antibodies in dogs (30.6%) and wild rats (9.5%) expands the potential host range of GII.11 PorNoV. Previous studies have documented HuNoVs infections in dogs (Charoenkul et al., 2020) and rats (Summa et al., 2018), suggesting bidirectional transmission risks. These animals, often living in close association with humans, could act as bridging hosts, facilitating spillover events. However, the mechanisms underlying cross-species transmission remain unclear, necessitating further investigation into the ecological and molecular factors driving these interactions.

Epitope analysis provided key insights into the antigenic relationships between GII.11 PorNoV and other NoV genotypes, identifying conserved sites and epitopes that may influence cross-species transmission. Among 35 analyzed P domain sequences, GII.11 PorNoV exhibited a 74.1% similarity with other GII genotypes, sharing epitopes H(P323) and I(W410/P513) with GI NoVs, and epitope I(Q409/W410) and HBGA binding site III(G450) with GII NoVs. However, critical mutations have led to a loss of HBGA binding capacity in GII.11 (Yang et al., 2019), suggesting alternative receptors might be involved in its infection process.

The weakly positive results observed for GII.6 and GII.13 NoV in the sera of hyperimmunized mice may potentially be attributed to the high antigen concentrations used during immunization. Specifically, mice hyperimmunized with repeated administrations of high-concentration antigens are likely to generate antibody titers significantly higher than those induced by natural infections (Chow et al., 2022; Hickey et al., 2023). To further investigate this phenomenon, paired human serum samples associated with outbreaks of GI.3 (Xie et al., 2020), GII.6 (Zhang et al., 2020), and GII.17 (Dai et al., 2017) NoV were tested to evaluate cross-reactivity between these genotypes and GII.11. None of the paired samples demonstrated seroconversion for GII.11, whereas antibody titers against the infecting genotypes increased by 4- to 64-fold. Additionally, with the exception of one convalescent-phase sample (sample GZ21 from the GII.17 outbreak), which exhibited weak positivity, all other samples tested negative for GII.11. These findings demonstrated the ability of the GII.11 NoV-LISA assay to effectively differentiate GII.11 from other NoV genotypes in real-world infection scenarios.

This study has several limitations that warrant consideration. Firstly, the human serum samples were sourced from a single community-based cohort in Jidong, which excluded those below the age of 18 years and limited their representativeness. Future research should expand sample collection to include diverse geographical locations, occupations, and demographic characteristics to better understand the seroprevalence of GII.11 PorNoV in humans. Secondly, only one human sample was strongly positive for GII.11 antibodies, with the remainder showing weak positivity, indicating a need for more comprehensive data to confirm these preliminary findings. Additionally, while the high seroprevalence in bats suggests their potential role as reservoirs, further ecological studies are required to clarify their involvement in viral maintenance and dissemination. Lastly, although epitope analysis revealed conserved HBGA binding sites, GII.11 PorNoV appears to have lost its HBGA binding capacity due to critical mutations, highlighting uncertainties in the mechanisms of host-virus interactions. Addressing these limitations through longitudinal studies and broader sampling will be crucial for understanding the zoonotic potential and transmission dynamics of GII.11 PorNoV.

## 5 Conclusion

In conclusion, this study challenges the traditional view of PorNoV as a swine-specific pathogen and provides compelling serological evidence for its cross-species transmission potential. The detection of GII.11 PorNoV-specific antibodies in humans and diverse animals underscores the complexity of NoVs ecology and the urgent need for interdisciplinary research to mitigate zoonotic risks. Future work should focus on elucidating transmission mechanisms, validating infectivity in non-porcine hosts, and assessing the clinical and epidemiological significance of these findings. Such efforts will be pivotal in shaping preventive strategies against emerging NoVs-related zoonoses.

## Data Availability

The raw data supporting the conclusions of this article will be made available by the authors, without undue reservation.

## Supplementary material

Supplementary Figure 1 Antigenic epitope analysis of GII.11 PorNoV. The protein sequences are named as: GenBank/host/genotype/year/isolate. The letters represent the corresponding amino acid abbreviations. Green, magenta, blue, and yellow filling colors indicate homology levels of 100%, ≤75%, ≤50%, and ≤33%, respectively. The line segments above the sequence denote different antigenic epitopes. Specifically, red, orange, yellow, green, cyan, blue, purple, brown, and black represent antigenic epitopes A–I, respectively. In addition, the pink color represents the histo-blood group antigen (HBGA) binding sites (HBS).
