# Supplementary material for: Detection of IgG antibody against the porcine norovirus GII.11 in human, domestic and wild animals
Source: Front Microbiol. 2025 Jun 26;16:1567132. doi: 10.3389/fmicb.2025.1567132 (PMC12240948; doi:10.3389/fmicb.2025.1567132)
Supplement: Supplementary file 1 [file Data_Sheet_1.pdf]

Supplementary Table 1 The raw data of Negative pig sera tested by GII.11 NoV-LISA

Sample	LU1	LU2	Mean±SD
Negative pig1	523	554	491.5±48.2
Negative pig2	498	504	
Negative pig3	427	443	

Note: Each sample was tested in duplicate to ensure reliability, with final results calculated as the mean of these measurements. The cut-off was set at 1,000 LU, defined as twice the mean LU of negative pig sera (491.5 LU, rounded to the nearest hundred).

Supplementary Table 2 The raw data of GII.11 NoV-LISA in cross-reactivity in hyperimmunized mice.

Sample	LU1	LU2	LU3	Mean±SD
GII.11	55569	54134	54969	54890.7±720.7
GI.2	559	538	513	536.7±23
GI.3	371	400	661	477.3±159.7
GI.5	386	391	434	403.7±26.4
GI.9	428	640	810	626±191.4
GII.2	582	462	504	516±60.9
GII.4	413	570	370	451±105.3
GII.6	1426	1422	1462	1436.7±22
GII.7	819	709	762	763.3±55
GII.12	945	904	932	927±21
GII.13	1334	1278	1253	1288.3±41.5
GII.17	756	622	758	712±77.9
GII.21	656	541	739	645.3±99.4
Rotavirus	541	416	424	460.3±70
Aichi virus	433	412	443	429.3±15.8
Negative	469	457	479	468.3±11

Note: Each serum was tested in triplicate, the results were presented as mean ± standard deviation (SD) in Figure 2A and 2B.

Supplementary Table 3 The raw data of GII.11 NoV-LISA in 250 Human sera.

**Sample**	**LU1**	**LU2**	**Mean±SD**	**Result**
JD1	535	466	500.5±48.8	Negative
JD2	528	524	526±2.8	Negative
JD3	1363	1291	1327±50.9	Positive
JD4	435	454	444.5±13.4	Negative
JD5	871	851	861±14.1	Negative
JD6	723	599	661±87.7	Negative
JD7	451	451	451±0	Negative
JD8	492	462	477±21.2	Negative
JD9	1391	1341	1366±35.4	Positive
JD10	726	736	731±7.1	Negative
JD11	2231	1997	2114±165.5	Positive
JD12	967	862	914.5±74.2	Negative
JD13	853	795	824±41	Negative
JD14	1408	1272	1340±96.2	Positive
JD15	712	698	705±9.9	Negative
JD16	550	515	532.5±24.7	Negative
JD17	1873	1808	1840.5±46	Positive
JD18	1043	948	995.5±67.2	Negative
JD19	847	842	844.5±3.5	Negative
JD20	744	704	724±28.3	Negative
JD21	679	697	688±12.7	Negative
JD22	828	783	805.5±31.8	Negative
JD23	735	678	706.5±40.3	Negative
JD24	490	557	523.5±47.4	Negative
JD25	1331	1238	1284.5±65.8	Positive
JD26	1586	1487	1536.5±70	Positive
JD27	622	575	598.5±33.2	Negative
JD28	528	557	542.5±20.5	Negative
JD29	433	358	395.5±53	Negative
JD30	498	540	519±29.7	Negative
JD31	1392	1364	1378±19.8	Positive
JD32	635	656	645.5±14.8	Negative
JD33	991	843	917±104.7	Negative
JD34	582	563	572.5±13.4	Negative
JD35	663	633	648±21.2	Negative
JD36	785	795	790±7.1	Negative
JD37	1410	1329	1369.5±57.3	Positive
JD38	647	546	596.5±71.4	Negative
JD39	950	983	966.5±23.3	Negative

JD40	1113	976	1044.5±96.9	Positive
JD41	1693	1547	1620±103.2	Positive
JD42	778	796	787±12.7	Negative
JD43	394	373	383.5±14.8	Negative
JD44	850	833	841.5±12	Negative
JD45	632	661	646.5±20.5	Negative
JD46	1108	884	996±158.4	Negative
JD47	553	496	524.5±40.3	Negative
JD48	823	706	764.5±82.7	Negative
JD49	977	952	964.5±17.7	Negative
JD50	648	613	630.5±24.7	Negative
JD51	933	882	907.5±36.1	Negative
JD52	865	776	820.5±62.9	Negative
JD53	973	872	922.5±71.4	Negative
JD54	325	294	309.5±21.9	Negative
JD55	1027	910	968.5±82.7	Negative
JD56	821	714	767.5±75.7	Negative
JD57	1146	1078	1112±48.1	Positive
JD58	1160	1107	1133.5±37.5	Positive
JD59	578	607	592.5±20.5	Negative
JD60	1313	1222	1267.5±64.3	Positive
JD61	444	456	450±8.5	Negative
JD62	691	583	637±76.4	Negative
JD63	557	574	565.5±12	Negative
JD64	1089	1007	1048±58	Positive
JD65	1204	1150	1177±38.2	Positive
JD66	1333	1266	1299.5±47.4	Positive
JD67	562	583	572.5±14.8	Negative
JD68	796	774	785±15.6	Negative
JD69	597	623	610±18.4	Negative
JD70	888	926	907±26.9	Negative
JD71	764	780	772±11.3	Negative
JD72	2090	2092	2091±1.4	Positive
JD73	1250	1141	1195.5±77.1	Positive
JD74	396	351	373.5±31.8	Negative
JD75	576	536	556±28.3	Negative
JD76	1564	1482	1523±58	Positive
JD77	799	741	770±41	Negative
JD78	597	520	558.5±54.4	Negative
JD79	1272	1135	1203.5±96.9	Positive
JD80	478	429	453.5±34.6	Negative

JD81	722	725	723.5±2.1	Negative
JD82	560	534	547±18.4	Negative
JD83	408	382	395±18.4	Negative
JD84	333	310	321.5±16.3	Negative
JD85	260	250	255±7.1	Negative
JD86	238	191	214.5±33.2	Negative
JD87	447	387	417±42.4	Negative
JD88	308	332	320±17	Negative
JD89	365	314	339.5±36.1	Negative
JD90	928	856	892±50.9	Negative
JD91	280	356	318±53.7	Negative
JD92	1134	1023	1078.5±78.5	Positive
JD93	1275	1216	1245.5±41.7	Positive
JD94	332	296	314±25.5	Negative
JD95	482	451	466.5±21.9	Negative
JD96	384	371	377.5±9.2	Negative
JD97	636	569	602.5±47.4	Negative
JD98	765	642	703.5±87	Negative
JD99	481	444	462.5±26.2	Negative
JD100	1025	970	997.5±38.9	Negative
JD101	687	618	652.5±48.8	Negative
JD102	610	532	571±55.2	Negative
JD103	483	466	474.5±12	Negative
JD104	1014	1005	1009.5±6.4	Positive
JD105	2295	2205	2250±63.6	Positive
JD106	1003	961	982±29.7	Negative
JD107	517	514	515.5±2.1	Negative
JD108	731	744	737.5±9.2	Negative
JD109	511	460	485.5±36.1	Negative
JD110	575	523	549±36.8	Negative
JD111	547	523	535±17	Negative
JD112	1292	1269	1280.5±16.3	Positive
JD113	905	912	908.5±4.9	Negative
JD114	2082	2100	2091±12.7	Positive
JD115	476	437	456.5±27.6	Negative
JD116	724	655	689.5±48.8	Negative
JD117	431	456	443.5±17.7	Negative
JD118	328	331	329.5±2.1	Negative
JD119	296	270	283±18.4	Negative
JD120	865	816	840.5±34.6	Negative
JD121	644	538	591±75	Negative

JD122	337	297	317±28.3	Negative
JD123	355	340	347.5±10.6	Negative
JD124	277	242	259.5±24.7	Negative
JD125	215	208	211.5±4.9	Negative
JD126	461	489	475±19.8	Negative
JD127	252	269	260.5±12	Negative
JD128	212	202	207±7.1	Negative
JD129	168	159	163.5±6.4	Negative
JD130	306	254	280±36.8	Negative
JD131	136	138	137±1.4	Negative
JD132	326	297	311.5±20.5	Negative
JD133	334	346	340±8.5	Negative
JD134	294	305	299.5±7.8	Negative
JD135	397	356	376.5±29	Negative
JD136	264	238	251±18.4	Negative
JD137	375	332	353.5±30.4	Negative
JD138	264	260	262±2.8	Negative
JD139	208	215	211.5±4.9	Negative
JD140	201	185	193±11.3	Negative
JD141	280	285	282.5±3.5	Negative
JD142	349	280	314.5±48.8	Negative
JD143	399	403	401±2.8	Negative
JD144	404	329	366.5±53	Negative
JD145	208	205	206.5±2.1	Negative
JD146	200	169	184.5±21.9	Negative
JD147	384	446	415±43.8	Negative
JD148	247	204	225.5±30.4	Negative
JD149	316	321	318.5±3.5	Negative
JD150	269	256	262.5±9.2	Negative
JD151	205	187	196±12.7	Negative
JD152	188	207	197.5±13.4	Negative
JD153	448	435	441.5±9.2	Negative
JD154	183	172	177.5±7.8	Negative
JD155	200	194	197±4.2	Negative
JD156	1393	1348	1370.5±31.8	Positive
JD157	369	327	348±29.7	Negative
JD158	506	510	508±2.8	Negative
JD159	222	265	243.5±30.4	Negative
JD160	814	736	775±55.2	Negative
JD161	407	380	393.5±19.1	Negative
JD162	722	689	705.5±23.3	Negative

JD163	464	446	455±12.7	Negative
JD164	858	689	773.5±119.5	Negative
JD165	3206	2740	2973±329.5	Positive
JD166	681	562	621.5±84.1	Negative
JD167	355	306	330.5±34.6	Negative
JD168	344	294	319±35.4	Negative
JD169	276	275	275.5±0.7	Negative
JD170	244	248	246±2.8	Negative
JD171	232	209	220.5±16.3	Negative
JD172	520	490	505±21.2	Negative
JD173	3891	3602	3746.5±204.4	Positive
JD174	446	412	429±24	Negative
JD175	537	448	492.5±62.9	Negative
JD176	522	485	503.5±26.2	Negative
JD177	396	388	392±5.7	Negative
JD178	412	396	404±11.3	Negative
JD179	326	301	313.5±17.7	Negative
JD180	1031	875	953±110.3	Negative
JD181	465	392	428.5±51.6	Negative
JD182	356	300	328±39.6	Negative
JD183	590	520	555±49.5	Negative
JD184	535	522	528.5±9.2	Negative
JD185	882	770	826±79.2	Negative
JD186	340	298	319±29.7	Negative
JD187	752	698	725±38.2	Negative
JD188	365	327	346±26.9	Negative
JD189	337	275	306±43.8	Negative
JD190	455	378	416.5±54.4	Negative
JD191	530	439	484.5±64.3	Negative
JD192	388	358	373±21.2	Negative
JD193	542	522	532±14.1	Negative
JD194	333	301	317±22.6	Negative
JD195	295	278	286.5±12	Negative
JD196	366	322	344±31.1	Negative
JD197	398	348	373±35.4	Negative
JD198	464	425	444.5±27.6	Negative
JD199	313	286	299.5±19.1	Negative
JD200	492	445	468.5±33.2	Negative
JD201	296	272	284±17	Negative
JD202	371	340	355.5±21.9	Negative
JD203	307	284	295.5±16.3	Negative

JD204	345	289	317±39.6	Negative
JD205	1146	1026	1086±84.9	Positive
JD206	506	463	484.5±30.4	Negative
JD207	378	348	363±21.2	Negative
JD208	1771	1641	1706±91.9	Positive
JD209	5976	5514	5745±326.7	**Positive***
JD210	1617	1545	1581±50.9	Positive
JD211	426	371	398.5±38.9	Negative
JD212	404	387	395.5±12	Negative
JD213	559	548	553.5±7.8	Negative
JD214	1510	1361	1435.5±105.4	Positive
JD215	250	227	238.5±16.3	Negative
JD216	655	609	632±32.5	Negative
JD217	428	386	407±29.7	Negative
JD218	1353	1188	1270.5±116.7	Positive
JD219	444	389	416.5±38.9	Negative
JD220	792	682	737±77.8	Negative
JD221	383	326	354.5±40.3	Negative
JD222	971	876	923.5±67.2	Negative
JD223	971	857	914±80.6	Negative
JD224	414	371	392.5±30.4	Negative
JD225	527	483	505±31.1	Negative
JD226	437	435	436±1.4	Negative
JD227	303	309	306±4.2	Negative
JD228	665	631	648±24	Negative
JD229	2267	2037	2152±162.6	Positive
JD230	486	465	475.5±14.8	Negative
JD231	433	394	413.5±27.6	Negative
JD232	511	514	512.5±2.1	Negative
JD233	390	367	378.5±16.3	Negative
JD234	380	335	357.5±31.8	Negative
JD235	1147	1079	1113±48.1	Positive
JD236	442	397	419.5±31.8	Negative
JD237	397	341	369±39.6	Negative
JD238	659	537	598±86.3	Negative
JD239	749	693	721±39.6	Negative
JD240	694	659	676.5±24.7	Negative
JD241	970	955	962.5±10.6	Negative
JD242	169	153	161±11.3	Negative
JD243	322	330	326±5.7	Negative
JD244	358	342	350±11.3	Negative

JD245	646	645	645.5±0.7	Negative
JD246	399	373	386±18.4	Negative
JD247	412	378	395±24	Negative
JD248	414	404	409±7.1	Negative
JD249	416	377	396.5±27.6	Negative
JD250	241	283	262±29.7	Negative

Note: Each sample was tested in duplicate to ensure reliability, with final results calculated as the mean of these measurements. **Positive*** represents strongly positive, with the mean LU value exceeding 4 times the cutoff (4,000 LU).

Supplementary Table 4 The raw data of GII.11 NoV-LISA in 142 Pig sera.

**Sample**	**LU1**	**LU2**	**Mean±SD**	**Result**
Pig-1	717	783	750±46.7	Negative
Pig-2	1599	1607	1603±5.7	Positive
Pig-3	2394	2437	2415.5±30.4	Positive
Pig-4	1676	1787	1731.5±78.5	Positive
Pig-5	1985	2097	2041±79.2	Positive
Pig-6	2131	2272	2201.5±99.7	Positive
Pig-7	1831	1811	1821±14.1	Positive
Pig-8	1116	1207	1161.5±64.3	Positive
Pig-9	1252	1383	1317.5±92.6	Positive
Pig-10	1072	1144	1108±50.9	Positive
Pig-11	1363	1392	1377.5±20.5	Positive
Pig-12	851	905	878±38.2	Negative
Pig-13	830	938	884±76.4	Negative
Pig-14	1101	1229	1165±90.5	Positive
Pig-15	1218	1248	1233±21.2	Positive
Pig-16	979	967	973±8.5	Negative
Pig-17	978	951	964.5±19.1	Negative
Pig-18	1495	1494	1494.5±0.7	Positive
Pig-19	1689	1699	1694±7.1	Positive
Pig-20	1020	1015	1017.5±3.5	Positive
Pig-21	1391	1457	1424±46.7	Positive
Pig-22	1155	1133	1144±15.6	Positive
Pig-23	1512	1476	1494±25.5	Positive
Pig-24	1144	1193	1168.5±34.6	Positive
Pig-25	3024	3016	3020±5.7	Positive
Pig-26	909	851	880±41	Negative
Pig-27	1071	1126	1098.5±38.9	Positive
Pig-28	829	829	829±0	Negative
Pig-29	1087	1177	1132±63.6	Positive
Pig-30	811	833	822±15.6	Negative
Pig-31	823	892	857.5±48.8	Negative
Pig-32	1566	1541	1553.5±17.7	Positive
Pig-33	2551	2534	2542.5±12	Positive
Pig-34	1055	1067	1061±8.5	Positive
Pig-35	1043	1047	1045±2.8	Positive
Pig-36	1115	1049	1082±46.7	Positive
Pig-37	1961	2037	1999±53.7	Positive
Pig-38	2114	2087	2100.5±19.1	Positive
Pig-39	2002	1964	1983±26.9	Positive

Pig-40	2915	3014	2964.5±70	Positive
Pig-41	1066	998	1032±48.1	Positive
Pig-42	762	773	767.5±7.8	Negative
Pig-43	1911	1945	1928±24	Positive
Pig-44	936	948	942±8.5	Negative
Pig-45	1220	1216	1218±2.8	Positive
Pig-46	8256	8530	8393±193.7	**Positive***
Pig-47	862	850	856±8.5	Negative
Pig-48	885	964	924.5±55.9	Negative
Pig-49	13328	13634	13481±216.4	**Positive***
Pig-50	1481	1555	1518±52.3	Positive
Pig-51	1489	1561	1525±50.9	Positive
Pig-52	6562	6667	6614.5±74.2	**Positive***
Pig-53	1512	1455	1483.5±40.3	Positive
Pig-54	4405	4247	4326±111.7	**Positive***
Pig-55	1937	1942	1939.5±3.5	Positive
Pig-56	1018	1058	1038±28.3	Positive
Pig-57	696	693	694.5±2.1	Negative
Pig-58	798	868	833±49.5	Negative
Pig-59	10585	10731	10658±103.2	**Positive***
Pig-60	5171	5174	5172.5±2.1	**Positive***
Pig-61	1880	1852	1866±19.8	Positive
Pig-62	3242	3335	3288.5±65.8	Positive
Pig-63	1525	1478	1501.5±33.2	Positive
Pig-64	2188	2146	2167±29.7	Positive
Pig-65	804	799	801.5±3.5	Negative
Pig-66	1663	1753	1708±63.6	Positive
Pig-67	1189	1296	1242.5±75.7	Positive
Pig-68	801	838	819.5±26.2	Negative
Pig-69	1528	1583	1555.5±38.9	Positive
Pig-70	1005	995	1000±7.1	Negative
Pig-71	1382	1356	1369±18.4	Positive
Pig-72	1044	1036	1040±5.7	Positive
Pig-73	626	656	641±21.2	Negative
Pig-74	768	829	798.5±43.1	Negative
Pig-75	5553	5669	5611±82	**Positive***
Pig-76	796	774	785±15.6	Negative
Pig-77	1073	1107	1090±24	Positive
Pig-78	1378	1345	1361.5±23.3	Positive
Pig-79	2741	2778	2759.5±26.2	Positive
Pig-80	1192	1262	1227±49.5	Positive

Pig-81	1265	1320	1292.5±38.9	Positive
Pig-82	1086	1155	1120.5±48.8	Positive
Pig-83	1086	1114	1100±19.8	Positive
Pig-84	3282	3313	3297.5±21.9	Positive
Pig-85	1140	1190	1165±35.4	Positive
Pig-86	1389	1316	1352.5±51.6	Positive
Pig-87	1938	1924	1931±9.9	Positive
Pig-88	3375	3507	3441±93.3	Positive
Pig-89	1028	1027	1027.5±0.7	Positive
Pig-90	1232	1312	1272±56.6	Positive
Pig-91	173	152	162.5±14.8	Negative
Pig-92	102	98	100±2.8	Negative
Pig-93	569	523	546±32.5	Negative
Pig-94	262	232	247±21.2	Negative
Pig-95	574	552	563±15.6	Negative
Pig-96	324	319	321.5±3.5	Negative
Pig-97	363	317	340±32.5	Negative
Pig-98	257	246	251.5±7.8	Negative
Pig-99	251	266	258.5±10.6	Negative
Pig-100	158	137	147.5±14.8	Negative
Pig-101	160	119	139.5±29	Negative
Pig-102	532	541	536.5±6.4	Negative
Pig-103	353	321	337±22.6	Negative
Pig-104	228	191	209.5±26.2	Negative
Pig-105	219	188	203.5±21.9	Negative
Pig-106	152	163	157.5±7.8	Negative
Pig-107	185	140	162.5±31.8	Negative
Pig-108	250	282	266±22.6	Negative
Pig-109	128	116	122±8.5	Negative
Pig-110	495	418	456.5±54.4	Negative
Pig-111	238	252	245±9.9	Negative
Pig-112	249	205	227±31.1	Negative
Pig-113	178	204	191±18.4	Negative
Pig-114	640	558	599±58	Negative
Pig-115	151	152	151.5±0.7	Negative
Pig-116	259	270	264.5±7.8	Negative
Pig-117	305	331	318±18.4	Negative
Pig-118	950	841	895.5±77.1	Negative
Pig-119	334	321	327.5±9.2	Negative
Pig-120	117	92	104.5±17.7	Negative
Pig-121	2332	2242	2287±63.6	Positive

Pig-122	111	89	100±15.6	Negative
Pig-123	337	310	323.5±19.1	Negative
Pig-124	202	192	197±7.1	Negative
Pig-125	300	303	301.5±2.1	Negative
Pig-126	300	324	312±17	Negative
Pig-127	784	725	754.5±41.7	Negative
Pig-128	236	208	222±19.8	Negative
Pig-129	248	236	242±8.5	Negative
Pig-130	509	450	479.5±41.7	Negative
Pig-131	468	368	418±70.7	Negative
Pig-132	732	630	681±72.1	Negative
Pig-133	94	90	92±2.8	Negative
Pig-134	282	248	265±24	Negative
Pig-135	900	853	876.5±33.2	Negative
Pig-136	228	210	219±12.7	Negative
Pig-137	762	793	777.5±21.9	Negative
Pig-138	246	250	248±2.8	Negative
Pig-139	321	246	283.5±53	Negative
Pig-140	123	100	111.5±16.3	Negative
Pig-141	387	374	380.5±9.2	Negative
Pig-142	217	191	204±18.4	Negative

Note: Each sample was tested in duplicate to ensure reliability, with final results calculated as the mean of these measurements. **Positive*** represents strongly positive, with the mean LU value exceeding 4 times the cutoff (4,000 LU).

.

Supplementary Table 5 The raw data of GII.11 NoV-LISA in 72 Dog sera.

**Sample**	**LU1**	**LU2**	**Mean±SD**	**Result**
Dog-1	1840	1592	1716±175.4	Positive
Dog-2	535	449	492±60.8	Negative
Dog-3	158	188	173±21.2	Negative
Dog-4	384	336	360±33.9	Negative
Dog-5	413	381	397±22.6	Negative
Dog-6	402	355	378.5±33.2	Negative
Dog-7	329	323	326±4.2	Negative
Dog-8	230	204	217±18.4	Negative
Dog-9	815	767	791±33.9	Negative
Dog-10	1341	1290	1315.5±36.1	Positive
Dog-11	543	554	548.5±7.8	Negative
Dog-12	197	188	192.5±6.4	Negative
Dog-13	502	436	469±46.7	Negative
Dog-14	198	199	198.5±0.7	Negative
Dog-15	253	218	235.5±24.7	Negative
Dog-16	1135	1025	1080±77.8	Positive
Dog-17	1341	1216	1278.5±88.4	Positive
Dog-18	486	412	449±52.3	Negative
Dog-19	796	728	762±48.1	Negative
Dog-20	568	526	547±29.7	Negative
Dog-21	2659	2296	2477.5±256.7	Positive
Dog-22	308	303	305.5±3.5	Negative
Dog-23	421	392	406.5±20.5	Negative
Dog-24	582	533	557.5±34.6	Negative
Dog-25	1358	1207	1282.5±106.8	Positive
Dog-26	707	626	666.5±57.3	Negative
Dog-27	1330	1228	1279±72.1	Positive
Dog-28	1102	1004	1053±69.3	Positive
Dog-29	4023	3705	3864±224.9	Positive
Dog-30	2191	2157	2174±24	Positive
Dog-31	387	401	394±9.9	Negative
Dog-32	772	703	737.5±48.8	Negative
Dog-33	513	475	494±26.9	Negative
Dog-34	1621	1530	1575.5±64.3	Positive
Dog-35	1386	1201	1293.5±130.8	Positive
Dog-36	235	206	220.5±20.5	Negative
Dog-37	2231	1986	2108.5±173.2	Positive
Dog-38	946	891	918.5±38.9	Negative
Dog-39	405	392	398.5±9.2	Negative

Dog-40	918	842	880±53.7	Negative
Dog-41	2557	2276	2416.5±198.7	Positive
Dog-42	4221	3930	4075.5±205.8	**Positive***
Dog-43	512	458	485±38.2	Negative
Dog-44	401	347	374±38.2	Negative
Dog-45	627	618	622.5±6.4	Negative
Dog-46	1078	1080	1079±1.4	Positive
Dog-47	470	471	470.5±0.7	Negative
Dog-48	746	661	703.5±60.1	Negative
Dog-49	281	264	272.5±12	Negative
Dog-50	257	213	235±31.1	Negative
Dog-51	19035	17558	18296.5±1044.4	**Positive***
Dog-52	394	351	372.5±30.4	Negative
Dog-53	444	360	402±59.4	Negative
Dog-54	206	183	194.5±16.3	Negative
Dog-55	441	387	414±38.2	Negative
Dog-56	6052	5796	5924±181	**Positive***
Dog-57	509	448	478.5±43.1	Negative
Dog-58	2080	1718	1899±256	Positive
Dog-59	439	445	442±4.2	Negative
Dog-60	5682	5142	5412±381.8	**Positive***
Dog-61	592	557	574.5±24.7	Negative
Dog-62	490	430	460±42.4	Negative
Dog-63	865	789	827±53.7	Negative
Dog-64	238	237	237.5±0.7	Negative
Dog-65	665	599	632±46.7	Negative
Dog-66	402	338	370±45.3	Negative
Dog-67	394	357	375.5±26.2	Negative
Dog-68	2782	2578	2680±144.2	Positive
Dog-69	483	434	458.5±34.6	Negative
Dog-70	1622	1486	1554±96.2	Positive
Dog-71	782	734	758±33.9	Negative
Dog-72	641	604	622.5±26.2	Negative

Note: Each sample was tested in duplicate to ensure reliability, with final results calculated as the mean of these measurements. **Positive*** represents strongly positive, with the mean LU value exceeding 4 times the cutoff (4,000 LU).

Supplementary Table 6 The raw data of GII.11 NoV-LISA in 43 Bat sera.

**Sample**	**LU1**	**LU2**	**Mean±SD**	**Result**
Bat-1	838	707	772.5±92.6	Negative
Bat-2	252	269	260.5±12	Negative
Bat-3	446	383	414.5±44.5	Negative
Bat-4	272	298	285±18.4	Negative
Bat-5	289	252	270.5±26.2	Negative
Bat-6	245	223	234±15.6	Negative
Bat-7	322	329	325.5±4.9	Negative
Bat-8	482	439	460.5±30.4	Negative
Bat-9	620	519	569.5±71.4	Negative
Bat-10	383	357	370±18.4	Negative
Bat-11	377	310	343.5±47.4	Negative
Bat-12	1785	1601	1693±130.1	Positive
Bat-13	3902	3622	3762±198	Positive
Bat-14	990	1032	1011±29.7	Positive
Bat-15	5493	5458	5475.5±24.7	**Positive***
Bat-16	1262	1304	1283±29.7	Positive
Bat-17	1478	1393	1435.5±60.1	Positive
Bat-18	1963	1771	1867±135.8	Positive
Bat-19	2102	2016	2059±60.8	Positive
Bat-20	3246	3030	3138±152.7	Positive
Bat-21	1815	1765	1790±35.4	Positive
Bat-22	1694	1773	1733.5±55.9	Positive
Bat-23	1623	1575	1599±33.9	Positive
Bat-24	4122	4193	4157.5±50.2	**Positive***
Bat-25	2846	2790	2818±39.6	Positive
Bat-26	1139	1160	1149.5±14.8	Positive
Bat-27	1947	1933	1940±9.9	Positive
Bat-28	6118	5947	6032.5±120.9	**Positive***
Bat-29	2116	2104	2110±8.5	Positive
Bat-30	2525	2372	2448.5±108.2	Positive
Bat-31	1486	1435	1460.5±36.1	Positive
Bat-32	2556	2450	2503±75	Positive
Bat-33	706	650	678±39.6	Negative
Bat-34	5751	5789	5770±26.9	**Positive***
Bat-35	3471	3321	3396±106.1	Positive
Bat-36	6168	5801	5984.5±259.5	**Positive***
Bat-37	345	306	325.5±27.6	Negative
Bat-38	1977	1877	1927±70.7	Positive
Bat-39	532	593	562.5±43.1	Negative

Bat-40	2221	2215	2218±4.2	Positive
Bat-41	3109	2954	3031.5±109.6	Positive
Bat-42	614	604	609±7.1	Negative
Bat-43	4339	4449	4394±77.8	**Positive***

Note: Each sample was tested in duplicate to ensure reliability, with final results calculated as the mean of these measurements. **Positive*** represents strongly positive, with the mean LU value exceeding 4 times the cutoff (4,000 LU).

Supplementary Table 7 The raw data of GII.11 NoV-LISA in 220 Rat sera.

Sample	LU1	LU2	Mean±SD	Result
Rat-1	302	275	288.5±19.1	Negative
Rat-2	2605	2456	2530.5±105.4	Positive
Rat-3	445	487	466±29.7	Negative
Rat-4	864	833	848.5±21.9	Negative
Rat-5	791	791	791±0	Negative
Rat-6	3516	3458	3487±41	Positive
Rat-7	902	946	924±31.1	Negative
Rat-8	764	770	767±4.2	Negative
Rat-9	764	710	737±38.2	Negative
Rat-10	708	662	685±32.5	Negative
Rat-11	261	258	259.5±2.1	Negative
Rat-12	1240	1183	1211.5±40.3	Positive
Rat-13	298	314	306±11.3	Negative
Rat-14	637	654	645.5±12	Negative
Rat-15	294	329	311.5±24.7	Negative
Rat-16	1058	1019	1038.5±27.6	Positive
Rat-17	359	309	334±35.4	Negative
Rat-18	250	265	257.5±10.6	Negative
Rat-19	345	339	342±4.2	Negative
Rat-20	138	121	129.5±12	Negative
Rat-21	295	291	293±2.8	Negative
Rat-22	282	296	289±9.9	Negative
Rat-23	109	137	123±19.8	Negative
Rat-24	134	143	138.5±6.4	Negative
Rat-25	1673	1616	1644.5±40.3	Positive
Rat-26	408	470	439±43.8	Negative
Rat-27	150	139	144.5±7.8	Negative
Rat-28	224	191	207.5±23.3	Negative
Rat-29	190	175	182.5±10.6	Negative
Rat-30	135	139	137±2.8	Negative
Rat-31	125	108	116.5±12	Negative
Rat-32	1058	1031	1044.5±19.1	Positive
Rat-33	807	809	808±1.4	Negative
Rat-34	522	520	521±1.4	Negative
Rat-35	1158	1144	1151±9.9	Positive
Rat-36	313	297	305±11.3	Negative
Rat-37	145	95	120±35.4	Negative
Rat-38	143	143	143±0	Negative
Rat-39	162	173	167.5±7.8	Negative

Rat-40	186	205	195.5±13.4	Negative
Rat-41	306	280	293±18.4	Negative
Rat-42	197	209	203±8.5	Negative
Rat-43	293	228	260.5±46	Negative
Rat-44	2012	1951	1981.5±43.1	Positive
Rat-45	183	166	174.5±12	Negative
Rat-46	83	99	91±11.3	Negative
Rat-47	928	905	916.5±16.3	Negative
Rat-48	218	215	216.5±2.1	Negative
Rat-49	2435	2362	2398.5±51.6	Positive
Rat-50	1811	1755	1783±39.6	Positive
Rat-51	1116	1129	1122.5±9.2	Positive
Rat-52	156	170	163±9.9	Negative
Rat-53	797	832	814.5±24.7	Negative
Rat-54	576	604	590±19.8	Negative
Rat-55	1013	989	1001±17	Positive
Rat-56	109	123	116±9.9	Negative
Rat-57	226	256	241±21.2	Negative
Rat-58	147	153	150±4.2	Negative
Rat-59	87	93	90±4.2	Negative
Rat-60	138	112	125±18.4	Negative
Rat-61	101	127	114±18.4	Negative
Rat-62	379	360	369.5±13.4	Negative
Rat-63	305	271	288±24	Negative
Rat-64	122	128	125±4.2	Negative
Rat-65	155	172	163.5±12	Negative
Rat-66	258	269	263.5±7.8	Negative
Rat-67	2320	2307	2313.5±9.2	Positive
Rat-68	263	224	243.5±27.6	Negative
Rat-69	874	881	877.5±4.9	Negative
Rat-70	182	162	172±14.1	Negative
Rat-71	137	134	135.5±2.1	Negative
Rat-72	200	218	209±12.7	Negative
Rat-73	153	118	135.5±24.7	Negative
Rat-74	153	133	143±14.1	Negative
Rat-75	513	500	506.5±9.2	Negative
Rat-76	2052	2206	2129±108.9	Positive
Rat-77	176	172	174±2.8	Negative
Rat-78	229	245	237±11.3	Negative
Rat-79	232	193	212.5±27.6	Negative
Rat-80	157	182	169.5±17.7	Negative

Rat-81	211	226	218.5±10.6	Negative
Rat-82	163	173	168±7.1	Negative
Rat-83	164	192	178±19.8	Negative
Rat-84	851	776	813.5±53	Negative
Rat-85	127	165	146±26.9	Negative
Rat-86	107	137	122±21.2	Negative
Rat-87	184	165	174.5±13.4	Negative
Rat-88	271	294	282.5±16.3	Negative
Rat-89	136	155	145.5±13.4	Negative
Rat-90	191	200	195.5±6.4	Negative
Rat-91	209	202	205.5±4.9	Negative
Rat-92	468	473	470.5±3.5	Negative
Rat-93	639	625	632±9.9	Negative
Rat-94	238	272	255±24	Negative
Rat-95	347	360	353.5±9.2	Negative
Rat-96	230	235	232.5±3.5	Negative
Rat-97	5197	5147	5172±35.4	**Positive***
Rat-98	265	263	264±1.4	Negative
Rat-99	470	417	443.5±37.5	Negative
Rat-100	278	260	269±12.7	Negative
Rat-101	325	330	327.5±3.5	Negative
Rat-102	503	486	494.5±12	Negative
Rat-103	605	575	590±21.2	Negative
Rat-104	383	408	395.5±17.7	Negative
Rat-105	261	300	280.5±27.6	Negative
Rat-106	933	871	902±43.8	Negative
Rat-107	365	357	361±5.7	Negative
Rat-108	2155	2040	2097.5±81.3	Positive
Rat-109	559	558	558.5±0.7	Negative
Rat-110	256	234	245±15.6	Negative
Rat-111	464	445	454.5±13.4	Negative
Rat-112	499	497	498±1.4	Negative
Rat-113	383	369	376±9.9	Negative
Rat-114	184	202	193±12.7	Negative
Rat-115	247	231	239±11.3	Negative
Rat-116	350	358	354±5.7	Negative
Rat-117	163	185	174±15.6	Negative
Rat-118	787	724	755.5±44.5	Negative
Rat-119	688	673	680.5±10.6	Negative
Rat-120	465	422	443.5±30.4	Negative
Rat-121	756	684	720±50.9	Negative

Rat-122	326	298	312±19.8	Negative
Rat-123	361	372	366.5±7.8	Negative
Rat-124	560	529	544.5±21.9	Negative
Rat-125	1108	1050	1079±41	Positive
Rat-126	304	276	290±19.8	Negative
Rat-127	155	134	144.5±14.8	Negative
Rat-128	207	163	185±31.1	Negative
Rat-129	154	160	157±4.2	Negative
Rat-130	226	196	211±21.2	Negative
Rat-131	181	145	163±25.5	Negative
Rat-132	144	108	126±25.5	Negative
Rat-133	206	187	196.5±13.4	Negative
Rat-134	890	872	881±12.7	Negative
Rat-135	279	232	255.5±33.2	Negative
Rat-136	330	341	335.5±7.8	Negative
Rat-137	202	199	200.5±2.1	Negative
Rat-138	211	219	215±5.7	Negative
Rat-139	142	159	150.5±12	Negative
Rat-140	150	131	140.5±13.4	Negative
Rat-141	208	168	188±28.3	Negative
Rat-142	517	502	509.5±10.6	Negative
Rat-143	917	832	874.5±60.1	Negative
Rat-144	211	194	202.5±12	Negative
Rat-145	349	334	341.5±10.6	Negative
Rat-146	398	336	367±43.8	Negative
Rat-147	258	260	259±1.4	Negative
Rat-148	186	144	165±29.7	Negative
Rat-149	235	241	238±4.2	Negative
Rat-150	485	443	464±29.7	Negative
Rat-151	132	116	124±11.3	Negative
Rat-152	131	120	125.5±7.8	Negative
Rat-153	263	303	283±28.3	Negative
Rat-154	343	315	329±19.8	Negative
Rat-155	274	290	282±11.3	Negative
Rat-156	1438	1397	1417.5±29	Positive
Rat-157	146	112	129±24	Negative
Rat-158	261	272	266.5±7.8	Negative
Rat-159	220	220	220±0	Negative
Rat-160	235	220	227.5±10.6	Negative
Rat-161	333	290	311.5±30.4	Negative
Rat-162	375	312	343.5±44.5	Negative

Rat-163	388	367	377.5±14.8	Negative
Rat-164	724	678	701±32.5	Negative
Rat-165	647	569	608±55.2	Negative
Rat-166	368	350	359±12.7	Negative
Rat-167	334	311	322.5±16.3	Negative
Rat-168	139	110	124.5±20.5	Negative
Rat-169	566	478	522±62.2	Negative
Rat-170	162	146	154±11.3	Negative
Rat-171	308	255	281.5±37.5	Negative
Rat-172	128	167	147.5±27.6	Negative
Rat-173	641	563	602±55.2	Negative
Rat-174	300	271	285.5±20.5	Negative
Rat-175	327	318	322.5±6.4	Negative
Rat-176	298	194	246±73.5	Negative
Rat-177	163	165	164±1.4	Negative
Rat-178	367	326	346.5±29	Negative
Rat-179	381	378	379.5±2.1	Negative
Rat-180	182	166	174±11.3	Negative
Rat-181	403	351	377±36.8	Negative
Rat-182	303	330	316.5±19.1	Negative
Rat-183	135	135	135±0	Negative
Rat-184	175	186	180.5±7.8	Negative
Rat-185	2578	2290	2434±203.6	Positive
Rat-186	219	169	194±35.4	Negative
Rat-187	111	90	100.5±14.8	Negative
Rat-188	144	126	135±12.7	Negative
Rat-189	307	344	325.5±26.2	Negative
Rat-190	381	352	366.5±20.5	Negative
Rat-191	284	298	291±9.9	Negative
Rat-192	497	447	472±35.4	Negative
Rat-193	1802	1835	1818.5±23.3	Positive
Rat-194	215	202	208.5±9.2	Negative
Rat-195	517	487	502±21.2	Negative
Rat-196	1543	1500	1521.5±30.4	Positive
Rat-197	205	215	210±7.1	Negative
Rat-198	220	205	212.5±10.6	Negative
Rat-199	143	133	138±7.1	Negative
Rat-200	171	142	156.5±20.5	Negative
Rat-201	321	312	316.5±6.4	Negative
Rat-202	273	276	274.5±2.1	Negative
Rat-203	537	494	515.5±30.4	Negative

Rat-204	139	135	137±2.8	Negative
Rat-205	112	110	111±1.4	Negative
Rat-206	112	60	86±36.8	Negative
Rat-207	166	147	156.5±13.4	Negative
Rat-208	319	343	331±17	Negative
Rat-209	293	306	299.5±9.2	Negative
Rat-210	156	160	158±2.8	Negative
Rat-211	292	244	268±33.9	Negative
Rat-212	139	138	138.5±0.7	Negative
Rat-213	225	214	219.5±7.8	Negative
Rat-214	192	181	186.5±7.8	Negative
Rat-215	189	163	176±18.4	Negative
Rat-216	183	182	182.5±0.7	Negative
Rat-217	183	198	190.5±10.6	Negative
Rat-218	324	257	290.5±47.4	Negative
Rat-219	284	258	271±18.4	Negative
Rat-220	156	147	151.5±6.4	Negative

Note: Each sample was tested in duplicate to ensure reliability, with final results calculated as the mean of these measurements. **Positive*** represents strongly positive, with the mean LU value exceeding 4 times the cutoff (4,000 LU).
