# Supplementary figures and images for: Detection of IgG antibody against the porcine norovirus GII.11 in human, domestic and wild animals
Source: Front Microbiol. 2025 Jun 26;16:1567132. doi: 10.3389/fmicb.2025.1567132 (PMC12240948; doi:10.3389/fmicb.2025.1567132)

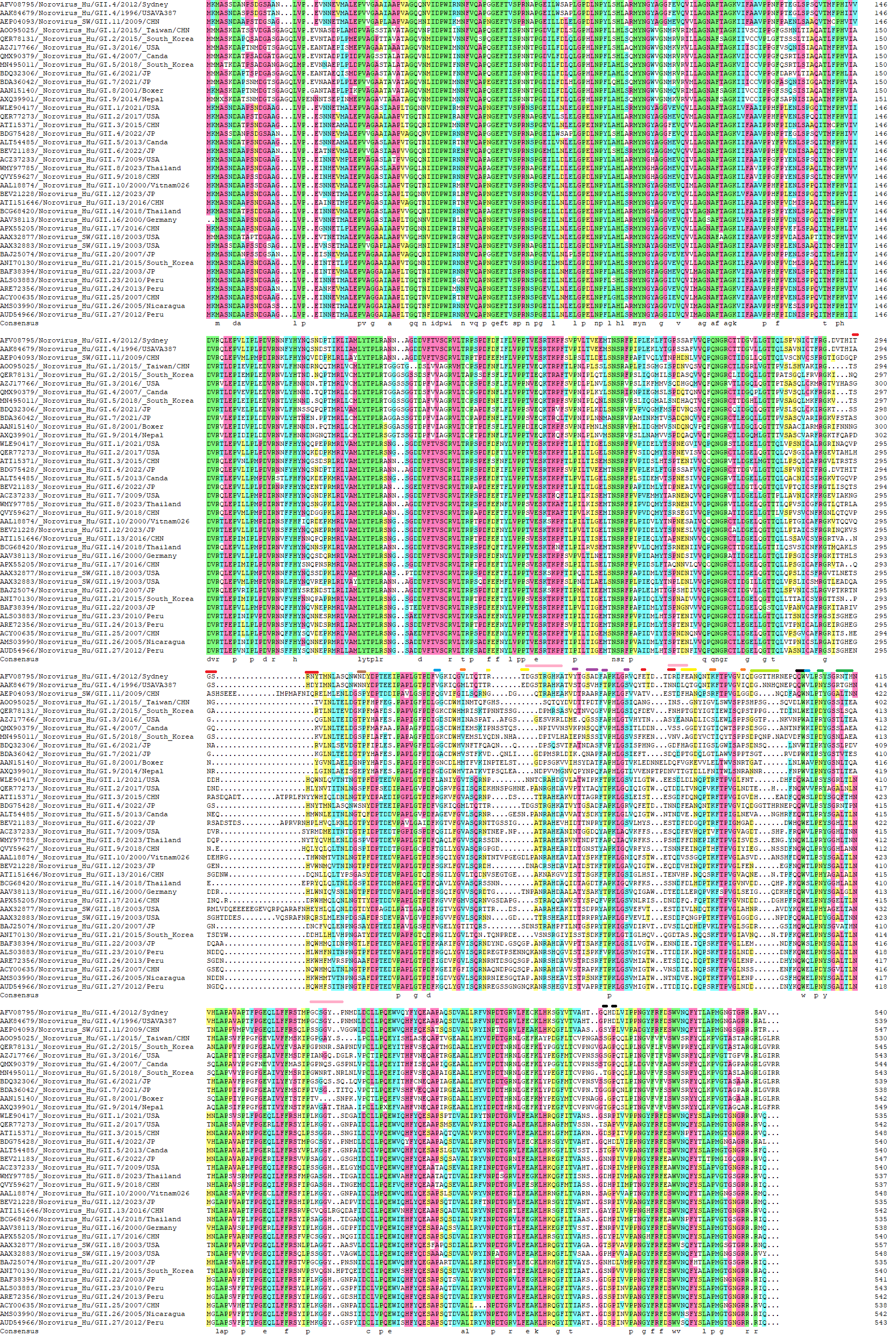

Supplement: Supplementary Figure 1 — Antigenic epitope analysis of GII.11 PorNoV. The protein sequences are named as: GenBank/host/genotype/year/isolate. The letters represent the corresponding amino acid abbreviations. Green, magenta, blue, and yellow filling colors indicate homology levels of 100%, ≤75%, ≤50%, and ≤33%, respectively. The line segments above the sequence denote different antigenic epitopes. Specifically, red, orange, yellow, green, cyan, blue, purple, brown, and black represent antigenic epitopes A–I, respectively. In addition, the pink color represents the histo-blood group antigen (HBGA) binding sites (HBS). [file Image_1.tif]
